# Institutionalised distrust and human oversight of artificial intelligence: towards a democratic design of AI governance under the European Union AI Act

**DOI:** 10.1007/s00146-023-01777-z

**Published:** 2023-10-06

**Authors:** Johann Laux

**Affiliations:** British Academy Postdoctoral Fellow, Oxford Internet Institute, University of Oxford, 1 St Giles’, Oxford, OX1 3JS UK

**Keywords:** Artificial intelligence, Human oversight, AI Act, Trust, Distrust, Institutional design

## Abstract

Human oversight has become a key mechanism for the governance of artificial intelligence (“AI”). Human overseers are supposed to increase the accuracy and safety of AI systems, uphold human values, and build trust in the technology. Empirical research suggests, however, that humans are not reliable in fulfilling their oversight tasks. They may be lacking in competence or be harmfully incentivised. This creates a challenge for human oversight to be effective. In addressing this challenge, this article aims to make three contributions. First, it surveys the emerging laws of oversight, most importantly the European Union’s Artificial Intelligence Act (“AIA”). It will be shown that while the AIA is concerned with the competence of human overseers, it does not provide much guidance on how to achieve effective oversight and leaves oversight obligations for AI developers underdefined. Second, this article presents a novel taxonomy of human oversight roles, differentiated along whether human intervention is constitutive to, or corrective of a decision made or supported by an AI. The taxonomy allows to propose suggestions for improving effectiveness tailored to the type of oversight in question. Third, drawing on scholarship within democratic theory, this article formulates six normative principles which institutionalise distrust in human oversight of AI. The institutionalisation of distrust has historically been practised in democratic governance. Applied for the first time to AI governance, the principles anticipate the fallibility of human overseers and seek to mitigate them at the level of institutional design. They aim to directly increase the trustworthiness of human oversight and to indirectly inspire well-placed trust in AI governance.

## Introduction

Human oversight of artificial intelligence (“AI”) has become a key 1mechanism of AI governance. Article 14 of the European Commission’s AI Act proposal (“AIA”) requires that AI systems are designed and developed such that they can be “effectively overseen by natural persons” (European Commission, 2021). Humans may be placed “in” or “on” the loop of an AI system, depending on whether they are involved in every decision the system makes or monitor the system’s overall operations (High-Level Expert Group on Artificial Intelligence 2019: 16). Various rationales for implementing human oversight exist. First, humans supposedly improve the performance and safety of an AI system (Brennan-Marquez et al. 2019: 746). Second, human oversight promotes values such as legitimacy, accountability, dignity, as well as human autonomy and agency (Brennan-Marquez et al. 2019: 746; Davidovic 2023; European Commission 2020: 9; High-Level Expert Group on Artificial Intelligence 2019: 16). According to Article 14(2) AIA, keeping humans involved aims at “preventing or minimising the risks to health, safety or fundamental rights”, thus addressing both safety and values. The AIA adds a third rationale: its overarching aim is to create European Union (“EU”) citizens’ trust in AI (European Commission 2021: 1). While empirically people’s trust will likely correlate with AI’s safety and impact on values (Langer et al. 2022), from a regulatory perspective trust is an end in itself: if people trust a technology, they are more likely to use it and hence to unlock its alleged economic and social potential (cf. [Laux et. al 2023b]). Empirical research suggests that people initially trust an AI system more if they are told that a human is in the loop (Aoki 2021). Yet, if this human’s oversight is not effective, then people’s trust in the AI system is not well placed and, potentially, harmful to their interests and wellbeing.

How to implement effective human oversight of AI is thus one of the big regulatory questions of our time. A recent survey of human oversight policies draws on empirical research to conclude that humans are mostly unable to perform their assigned oversight functions (Green 2022). Humans have been shown to both over-rely (“automation bias”) and under-rely (“algorithm aversion”) on algorithmic advice and fare badly at judging the accuracy of algorithmic predictions (Green 2022: 7; Jones-Jang and Park 2022). The empirical record of such biases is still subject to debate (see the references in: (Hacker 2022: 19)). However, in computer-system design, it is common to consider when humans are best kept “out of the loop” (Cranor 2008: 1). In aviation, manual operation by humans has been increasingly limited and replaced by automation precisely out of safety concerns (Banks et al. 2019: 256). Commanding human oversight by law is thus no panacea for defusing AI’s risks. To be effective, it requires careful calibration.

It has been argued before that the challenge of effective human oversight is best addressed at the level of institutional design (Green 2022). This paper follows this approach, adds novel analytical tools and design principles to its repertoire, and demonstrates its practical impact by reference to the EU’s emerging regulatory order for AI. In doing so, this paper aims to make three contributions to the budding field of research on AI governance. The first contribution is analytical. It surveys the emerging laws of human oversight in the EU, especially the proposed AIA (Sect. 2). The AIA is the first comprehensive law to address the risks of AI and may become a global standard. This article reads it as a bellwether of the evolving regulatory landscape without, however, tying its propositions to the AIA’s particular norms. It will be shown that the AIA requires oversight to be effective without providing much guidance on how effectiveness can be achieved and leaves oversight obligations for AI developers underdefined. At the time of writing, both the Council (Council of the European Union 2022) and the European Parliament (European Parliament 2023) have proposed amendments to the Commission’s proposal. The final text of the AIA has not yet been decided on. Below, “AIA” refers to the Commission’s proposal, and “AIA*Council” and “AIA*Parliament” to the respective amendments.

The second contribution is also analytical. It presents a novel taxonomy of human oversight roles, differentiated along two types of influence on the decisional output of an AI system: first-degree and second-degree oversight. First-degree overseers will be understood as having counterfactual influence on a decision made or supported by an AI. Having counterfactual influence means that the initial output of an AI system could have been different due to human involvement (cf. the scenario developed in (Hacker 2022: 36)). First-degree overseers will often be local decision-makers whose work is supported by the AI through predictions or recommendations. Second-degree overseers are one step removed from the local decision in question, fulfilling auditing or review roles. They have no counterfactual influence on the initial decisional output but instead are corrective of it. Simply put, first-degree overseers will regularly be involved during the AI-supported decision-making, second-degree overseers will act *ex post*, for example by checking system logs. Both types face challenges of lacking competence and false incentives (Sect. 4). The two types of oversight and the two challenges create a two-by-two matrix with four distinct areas of concern (see Table 2). The taxonomy thus allows to propose solutions to the problem of effectiveness which are tailored to the type of oversight in question.

The third contribution is theoretical. It makes the novel suggestion to utilise “institutionalised distrust” to address the four identified areas of concern (Sect. 5). Stemming from scholarship within democratic theory (Braithwaite 1998; Sztompka 2000), the “institutionalisation of distrust in the architecture of democracy” has been argued to provide a governance order in which people’s well-placed trust can emerge (Sztompka 2000: 12). Sztompka calls this the “paradox of democracy” (Sztompka 2000: 12): precisely because foundational principles of democracy imply some degree of institutionalised distrust, trust within governmental environments can emerge. Applied to AI governance, distrust in the abilities and motivations of human overseers should be utilised for the institutional design of their roles. This would allow the cultivation of well-placed (instead of naïve) trust in AI systems. This article formulates six principles for the institutionalisation of distrust in human oversight: justification; periodical mandates; collective decisions; limited competence of institutions; justiciability and accountability; transparency. Led by democratic theory, the principles developed in this article apply first and foremost to public institutions but can still be relevant for governing AI in the private sector. Of course, institutionalising distrust in human oversight would not guarantee risk-free AI systems. Instead, it would provide a general scaffolding along which trustworthy local implementations of human oversight may be built.

Lastly, the term “AI” is used here not in a particular technical understanding, but following the definition of Article 3 AIA*Parliament, i.e. as a “machine-based system that is designed to operate with varying levels of autonomy and that can […] generate outputs such as predictions, recommendations, or decisions that influence physical or virtual environments.”

## The emerging laws of oversight

Article 14(1) AIA explicitly demands effective human oversight for high-risk AI systems before these can be placed on the market. Article 29 AIA states that the implementation of human oversight measures is an obligation for users of high-risk AI. This is substantively more than just nominal human involvement and upgrades current standards in EU data protection law (Green 2022: 4). Article 22(1) General Data Protection Directive (“GDPR”) gives data subjects the right to not be subject to solely automated decision-making without consent. The narrow focus on “solely” automated decisions has led to reasonable criticism (cf. (Wachter et al. 2017)). First, AI systems making decisions without any kind of human involvement could remain rare instances, thus drastically narrowing the scope of Article 22 GDPR (Veale and Edwards 2018: 400). Often, AI will assist humans by providing predictions, enhancing their decision-making (Agrawal et al. 2022: 9). For example, an AI-driven recommender system utilised in the admission of students would thus be excluded from the scope of Article 22 GDPR if the ultimate decision about admission is left to a human employee (Wendehorst 2021: 58–59). Second, if even nominal human involvement is enough to exclude an AI system from the reach of Article 22 GDPR, then there is a risk of humans merely “rubber stamping” the use of AI without any substantial oversight (Veale and Edwards 2018: 400; Wachter et al. 2017: 88). The Article 29 Data Protection Working Party (“WP29”), therefore, emphasises that the data controller “must ensure that any oversight of the decision is meaningful, rather than just a token gesture” (Article 29 Data Protection Working Party 2017: 10).

In comparison, Article 14 AIA is more inclusive. Its scope does not differentiate according to the degree of human involvement, therefore, also covering recommender systems. However, Article 14 AIA only applies if the AI system is “high-risk” according to Article 6 AIA and Annex III AIA. Moreover, the Commission’s AIA proposal does not clarify when and how human oversight is required (Ebers et al. 2021: 596). Intuitively, one may argue that oversight pertains to the actual operation of a system, not its developmental stage.

Defining the scope of human oversight is not least important for delineating the obligations between AI providers and users/deployers. Generally, humans may influence the decisional output of an AI system during its entire lifecycle, i.e. before, during, or retrospectively after an AI system is put into service (for the AI lifecycle, see: (De Silva and Alahakoon 2022)). Article 14(1) AIA states that natural persons must oversee high-risk AI systems “during the period in which the AI system is in use”. Art 14(1) AIA*Parliament adds that “thorough investigation after an incident” must be possible. This suggests that the temporal scope of human oversight begins when the AI system is put into use and, potentially, retrospectively after it has been shut down following an incident. However, Article 14(3) AIA states that human oversight measures should be “identified” and (if possible) “built” into the AI system by the provider (i.e. developer) *before* a high-risk AI system is placed on the market or put into service. Article 29(2) AIA speaks of users “implementing” oversight measures which have been “indicated” by the provider. This establishes a shared responsibility between the developer, who provides for their AI to be oversee-able, and the user/deployer, who executes the oversight. Moreover, AI systems will often continue to evolve while being in use. They may be retrained with different data or incorporate user feedback. This can mean going back from the deployment to the development stage of an AI. In a continuously learning and changing AI system, the distinction between its developer and its user becomes blurry (cf. also: (Andrade and Zarra 2022: 33)). The technology is delivered dynamically and not as a static product or “one-off service” (Edwards 2022: 5). This suggests leaving the temporal scope of Article 14 AIA principally open to include all major stages of an AI lifecycle, including the design and development stages. Oversight responsibilities should fall on AI developers on a case-by-case basis and whenever needed to fill gaps in oversight which would otherwise emerge.

Furthermore, Article 14 AIA does not provide much information as to what will make human oversight effective or meaningful. Article 14(3) and (4) AIA outline vague systems-design measures aiming to give human overseers the ability to monitor and intervene in the AI’s decision-making. Humans in charge of oversight must be able to “understand the capacities and limitations” of an AI system, remain aware of “automation bias”, be able to “correctly interpret” an AI system’s output, be able to decide not to use an AI system or “disregard, override or reverse” its decisions, and “interrupt” the system’s operation (Art. 14(4) AIA). Article 14(4)(e) AIA*Parliament adds that humans should not interfere with an AI system if this “increases the risks or would negatively impact the performance.” Recital 48 AIA adds that human overseers must “have the necessary competence, training and authority to carry out that role.” Article 29(1)(a) AIA*Council repeats the point made in Recital 48 AIA nearly verbatim. Article 14(1) AIA*Parliament adds that human overseers should have a “sufficient level of AI literacy” and the “necessary support and authority”. The term “AI literacy” has been added by the Parliament in a new Article 4b. It is vaguely defined as an understanding of the “basic notions and skills about AI systems and their functioning, including the different types of products and uses, their risks and benefits” (Art. 14b(3) AIA*Parliament). Article 29(1a)(ii) AIA*Parliament states that human overseers must be “competent, properly qualified and trained, and have the necessary resources in order to ensure the effective supervision of the AI system.” In sum, the AIA requires human overseers to be sufficiently competent and authorised to intervene in an AI system. It does not clarify which exact qualifications (let alone certifications) those individuals must have which are tasked with executing Article 14 AIA functions. For a horizontal law such as the AI Act, and in absence of existing standards for effective oversight (cf. also (Hacker 2022: 36–37)), this may well be as much as is currently possible.

The requirements in the AIA do further not specify whether and if so, to what degree, human oversight may be augmented by AI. Some form of hybrid intelligence may be necessary for effective AI oversight, given the foreseeable challenges to human competence when auditing complex AI systems. Just consider the plethora of methods developed for explaining AI models (Vilone and Longo 2021: 615–616), some of which may themselves fall under the regulatory definition of “AI” (Gyevnar et al. 2023: 6). At least for high-risk systems, Article 14(4)(d) and (e) AIA suggest that humans (and not machines) must have ultimate authority over outcomes and be able to override an AI’s decision or recommendation for human oversight to be “effective”.^2^ However, the aforementioned Article 14(4)(e) AIA*Parliament demands to refrain from human interference if it increases risks or negatively impacts performance.

Whether or not to allow human override of automated decisions is hard to determine in the abstract. Even within concrete domains, the choice is not always clear. Take aviation as an example. Some aerospace manufacturers implemented “hard automation” in which an automated flight system can override human input to prevent human error (Banks et al. 2019: 256; Young et al. 2007). Other manufacturers opted for “soft automation”, allowing for automated recommendations or decisions to be overridden by a human operator (Banks et al. 2019: 256). Hard automation would *prima facie* be more difficult to reconcile with “effective” human oversight according to Article 14(4) AIA than soft automation. However, as the rationales for human oversight in the AIA acknowledge safety concerns, hard automation for some steps in a chain of decisions within a complex AI system could be compatible with Article 14(4) AIA, especially if the Parliament’s amendment will become law.

Lastly, technical standards will play a role in defining functions and methods of human oversight.^3^ In May 2023, the European Commission sent its request for standardisation to the European Committee for Standardisation (CEN) and the European Committee for Electrotechnical Standardisation (CENELEC) (European Commission, 2023b), including standards that “specify measures and procedures for human oversight” (European Commission, 2023a: 5). Meanwhile, international standardisation bodies have already begun to develop standards for AI. As of August 2023, the International Organization for Standards’ (“ISO”) subcommittee 42 (“SC 42”) published 20 standards on AI, including so-called technical reports and technical specifications.^4^ So far, the published standards do not provide further information on how to implement effective human oversight of AI. ISO/IEC TR 24028 (“overview of trustworthiness in artificial intelligence”) merely emphasises the importance of human decision-makers who possess the agency and autonomy to intervene in the final decision-making process (International Organization for Standardization (ISO) and International Electrotechnical Commission (IEC), 2020: 9.4.2). ISO/IEC TR 24368 (“overview of ethical and societal concerns”) requires high-risk AI systems to “have a qualified human in the loop to provide authorisation of automated decisions” (International Organization for Standardization (ISO) and International Electrotechnical Commission (IEC), 2022: 6.2.8).

In summary, the emerging regulatory landscape of human oversight so far demands human overseers to be competent and authorised to intervene in and even override AI systems, without further guidance on which competences are needed (beyond a vague description of AI literacy). Future technical standards will expectably provide best practices, measures, and procedures. Developers and users of AI systems will thus enjoy wide discretion in deciding how to institutionally implement their human oversight obligations. While many institutional design choices will depend on the local characteristics of an AI system, this article addresses issues of institutional design which are relevant for most AI oversight systems. As a first step of this horizontal approach, the following section introduces a novel typology of human oversight roles.

## First-degree and second-degree human oversight

The typology offered in this section differentiates the roles of human overseers depending on whether they have counterfactual influence on a decisional outcome of an AI system. Having counterfactual influence means that the initial output of an AI system could have been different due to human involvement (cf. the scenario developed in (Hacker 2022: 36)). Without counterfactual influence, humans can only step in to correct and reverse a decision after the initial output is produced. The examples in the following paragraphs will illustrate the distinction further.

Broadly speaking, AI systems can be fully or partially automated. In partially automated systems, AI will often provide decision support for professionals (Agrawal et al. 2022: 9; Veale and Edwards 2018: 400). In health care, for example, AI may deliver diagnostic predictions and treatment suggestions (Strickland 2019). In criminal law, judges may draw on algorithmic risk assessments of defendants (Angwin et al. 2016). At least formally, the final decision will then remain with the medical or judicial professional. Following a distinction by Agrawal et al., in such systems the AI provides the prediction and humans execute judgement, i.e. they determine the importance or value attached to the AI’s prediction (Agrawal et al. 2022: 147). Bracketing questions about its effectiveness, there is thus human oversight in partially automated AI systems that provide decision support because humans remain involved in the final decision-making. More so, humans in partially automated systems will regularly have counterfactual influence: the decisional output will depend on their judgement of the AI’s prediction. Here, human oversight is thus constituent to the outcome. We may call these cases “first-degree” human oversight.

First-degree overseers can be experts such as doctors and judges or professionals with light to moderate training (for the latter, see (Perrigo 2023)). At Meta, for example, removal of posts that violate the firm’s content policies appears to be largely automated, with just a smaller number of posts going to human content moderators for review (Meta 2022a, 2022b). In such cases, automated content review requires human input or has missed something it should have flagged and removed (Meta 2022a, 2022b). Flagging content for further human review is currently a major use case for machine learning: as human content moderation cannot be easily scaled to meet the need of content-hosting companies, machine learning systems pre-select a small fraction of content for human review (AWS, n.d.). As long as content moderators’ judgments are constitutive to the initial decision of removing a post (or keeping it online), their involvement qualifies as first-degree oversight.

Even in fully automated AI systems, humans still influence the decisional output. Building an AI model and developing its benchmarks for predictions or classifications has counterfactual influence on the AI’s outputs. Imagine AI developers setting threshold scores regarding, for example, when a driverless car should brake or when to reject a credit card payment (Agrawal et al. 2022: 155–165). How high the probability of fraud must be before a credit card payment gets rejected has counterfactual influence on the outcome. If it is higher, more transactions will be cancelled than if it is lower. However, once the threshold is set and the system is put in place fully automated, there is no further human judgement involved. Every transaction above the threshold will be cancelled. The baseline for these cancellations, however, are human judgments about threshold scores (Agrawal et al. 2022: 120). Besides finance, online advertising is another domain in which full automation has already been broadly implemented (Agrawal et al. 2022: 30–33; Veale and Edwards 2018: 400).

For fully automated systems, there are thus logically only two points in time when human oversight could be executed: before the AI system is implemented and after it has made a decision. However, if stepping in after a decision has been made, humans have no counterfactual influence on the initial decisional output of the AI. We may thus call these situations “second-degree” human oversight. Instead of being constituent, second-degree oversight is corrective of an AI’s decisional output. For fully automated systems, Article 22(3) GDPR mentions such second-degree oversight by referring to the data subject’s right to contest and the right to obtain human intervention (for a discussion of these rights, see: (Wachter et al. 2017: 93–96; Wendehorst 2021: 52–63)). Second-degree oversight is not exclusive to fully automated systems. A right to human review may become relevant for partially automated systems, too. The AIA applies to both fully and partially automated systems and envisions that conformity with its norms may be assessed through third-party audits (see further: (Mökander et al. 2022)). Moreover, Article 14(1) AIA*Parliament demands that the design of high-risk AI systems must at least allow for “thorough investigation” after the fact. Reviews and audits are forms of second-degree oversight, as the human overseers are not directly involved in the AI’s or AI-supported decision-making. They are one step removed from the process and have no counterfactual influence on the initial output of the system.

Second-degree oversight may further have a layered structure. At Meta, the above-mentioned first-degree oversight through human content moderators is itself overseen by the so-called Oversight Board. The Oversight Board provides an appeals process for content moderation decisions made on Facebook and Instagram, which are both owned by Meta (Oversight Board, n.d.). In 2022, for example, the Oversight Board decided to restore a post on Facebook which had previously been removed by the company’s partially automated content removal system (Colombian police cartoon 2022).

The distinction between first- and second-degree oversight adds analytical depth to normative demands for “meaningful” oversight. It has been stated before that meaningful oversight requires that someone has the “authority and competence to change the decision” (Article 29 Data Protection Working Party 2017: 10) or has the “ability to override an AI’s decision or recommendation” (Brennan-Marquez et al. 2019: 749). However, changing or overriding an AI output can have different normative consequences, depending on whether it is constitutive of a decision or corrective of it. It may, for example, matter in establishing who is liable for damages (cf. (Hacker 2022: 36)).

With a view on the emerging laws of oversight, the typology developed here raises two questions. First, do AI developers qualify as first-degree overseers? Analytically, developers will regularly have counterfactual influence on a system’s outputs, for example by determining classification thresholds or organisational workflows. For partially automated systems the question may be less decisive than for fully automated systems in which the developer’s judgments make up most (if not all) of the human input in a decision. Legally, our analysis in Sect. 2 showed that Article 14 AIA envisions a shared responsibility between the developer, who provides for their AI to be oversee-able, and the user/deployer, who executes the oversight. For fully automated systems, the user’s execution of human oversight might amount to nothing more than interrupting the system (i.e. pressing the “stop button”) to avoid harm. If this was enough to fulfil the demands of Article 14 AIA, then human oversight would amount to a very thin requirement. Following the criterion of counterfactual influence developed in this section, some oversight obligations should fall on AI developers if the dynamic evolution of their AI models would otherwise render oversight by the user largely ineffective.

Second, whether second-degree oversight falls under Article 14 AIA must remain an open question at the time of writing. If the above-mentioned amendment by the European Parliament is successful, ex post investigations must be feasible before a high-risk AI system can be placed on the market. Besides the AIA, the right to review in Article 22 GDPR guarantees second-degree oversight for fully automated AI-driven decisions within the remit of the GDPR.

Table 1 presents a two-by-two matrix of the two degrees of human oversight, listing AI development with asterisks to signify the need to decide on its inclusion on a case-by-case basis.

**Table 1:** First-degree and second-degree oversight

	First-degree oversight	Second-degree oversight
Fully automated system	*AI development	Reviews Audits Appeals
Partially automated system	*AI development AI as decision support	Reviews Audits Appeals

“*” signifies that inclusion needs to be decided case-by-case

## Two challenges for human oversight

Like all humans, overseers are fallible actors. Human oversight as a governance mechanisms can thus give a false sense of security if humans fail systematically at overseeing AI (cf. (Green 2022: 7)). A general assessment (or prediction) of the reliability of human overseers is currently unavailable. Take “automation bias” and “algorithm aversion” as examples, two opposing reactions to AI shown in humans (Jones-Jang and Park 2022: 2).

Algorithm aversion occurs when people prefer human predictions over algorithmic predictions even when the algorithm is shown to be more accurate (Dietvorst et al. 2015). The aversion has been evidenced in both lay people and experts (Dietvorst et al. 2015: 114). Due to automation bias, people may overestimate an AI’s accuracy and consistency in its performance (Jones-Jang and Park 2022: 2). Logg et al. showed that lay people adhere more to advice when they think it comes from an algorithm than from a person (Logg et al. 2019). The effect waned when participants had to choose between the algorithm’s advice and their own judgement and when they had expertise in forecasting (Logg et al. 2019). Applied to human oversight, this would suggest that untrained overseers may be more prone to automation bias and thus deferring their judgement to the algorithmic prediction than expert overseers. Untrained second-degree overseers may be more affected than untrained first-degree overseers if they were to decide a case of conflict in which they must choose between an AI’s prediction and that of a (first-degree) human. Expert human overseers may hurt their accuracy by over-relying on their own judgement versus the algorithm’s recommendation.

The point here is that even with our simplified taxonomy of oversight roles, we can adjust our expectations as to how much such biases affect either first- or second-degree overseers. At this level of abstraction, this may already be as good as it gets. Generalised assessments for AI as a technology which hold over a wide scope of AI applications will most likely remain unavailable. Investigations into local use cases, testing such cognitive biases as mentioned above and their effects on human oversight on a particular AI system are, therefore, necessary (see Sect. 5.2).

Human overseers may further lack the appropriate training to understand the functioning of an AI system. Generally speaking, the introduction of a novel AI system will regularly proceed whenever the AI outperforms humans at a given task (Green 2022: 11). Add empirical research such as the aforementioned work on automation bias to the picture, and some argue that overseeing automated systems is an impossible assignment for humans ((Green 2022: 11); citing: (Bainbridge 1983)). At a minimum, it raises the cognitive bar for humans who are supposed to intervene in an AI system to improve its reliability. Again, some commentators doubt that additional training can alleviate the gap between humans and AI in the expected average decisional quality, at least along some parameters ((Green 2022: 11–12); citing: (Parasuraman and Manzey 2010; Skitka et al. 1999)). Let us call this the challenge from lack of competence: humans may lack the skills and cognitive infallibility to effectively oversee AI systems.

Another challenge stems from incentive structures. Human overseers may lack the time to form a judgement which is not entirely based on the predictive input of the AI. Some may simply become tired and bored by their task ((Cranor 2008: 1); drawing on: (Flechais et al. 2005)). Financial or commercial incentives and self-interest can likewise result in subquality outcomes. With big tech companies leading the development of AI, auditors risk capture by industry interests to receive repeated auditing commissions (Laux et. al 2021). It is the objective of institutional design to remove obstacles for meaningful human oversight and to secure against misrule (Elster 2013: 1). It must, therefore, consider the motives (incentives) of human overseers and limit their ability to do harm (Elster 2013: 2). First- and second-degree overseers may face structurally different motivational environments, not least because of their different positioning within or outside an organisation. We can, therefore, adapt the two-by-two matrix from the previous section as follows (the letters A–D denote areas of concern which the next section addresses):

## Institutionalising distrust in AI oversight

To be itself trustworthy, human oversight of AI must address the challenges of lacking competence and false incentives outlined above. This article suggests that institutionalising distrust in human oversight considers both issues by providing a decidedly democratic governance structure for AI oversight. There is a longstanding tradition in democratic theory of optimising institutional design to prevent public decision-makers’ competence and motives from being led astray (cf. Bentham et al. 1990; Elster 2013)). Especially liberal democratic theorists have recurrently embraced distrust as being beneficial to democracy and the design of its institutions.^5^ This does not imply that citizens’ actual distrust of democratic institutions is favourable (Bertsou 2019: 216). Rather, the lens of distrust can help to create trustworthy institutions which, in turn, may inspire people’s trust in democracy.

This section aims to show that deriving principles of institutional design for human oversight from the lens of distrust can make a positive contribution to AI governance. As mentioned, the primary target of the principles outlined below are AI systems in the public sector. They may nevertheless resonate with private sector uses of AI.

### The democratic theory of institutionalised distrust

Sztompka describes how democracies are conducive to generating a “culture of trust […] due precisely to the institutionalization of distrust in the architecture of democracy” (Sztompka 2000: 12). The principles of democratic rule provide a “backup or insurance for those who would be ready to risk trust” (Sztompka 2000: 12). While Sztompka speaks of trust in an entire system of governance, institutionalising distrust in AI oversight proceeds within a more confined domain. Human oversight is one of many mechanisms of AI governance. At the same time, AI governance and oversight can influence the broader institutional setting. When a public institution implements a novel AI system, this can change its perceived trustworthiness and the processes by which it is able to generate citizens’ trust in its actions ([Laux et. al 2023b: 8]; drawing on: Bodó, 2021: 2675).

Sztompka considers a specific form of trust. His “culture of trust” is an empirical trait of human collectives, neither an individual’s psychological disposition nor a rationalistic calculation of the trustworthiness of the trustee (Sztompka 2000: 5–6). Generally, trust research is notoriously difficult to compare across and even within disciplines, as conceptions of trust vary widely ([Laux et. al 2023b: 6–9]). Hence, applying Sztompka’s notion of institutionalised distrust to the governance of AI requires some adjustment. First, we can differentiate normative accounts of trustworthiness from empirical measures of people’s trust. Placing too much trust in the normatively not-so-trustworthy is unjustified and, potentially, naïve. Second, empirical research suggests that trust in institutions will often be an important explanatory variable for the emergence of trust in a technology such as AI (cf. [Laux et. al 2023b: 17–23]). The AIA presents a regulatory framework for the development of “trustworthy AI” (AIA, p. 1). As (empirical) trust cannot be created on command, signalling (normative) trustworthiness is the most promising option for the stated aim of creating the level of trust needed for a broad uptake of AI in the EU ([Laux et. al 2023b: 1]). Thus, to institutionalise distrust means to increase the trustworthiness of a system of governance and, as Sztompka argues, to provide opportunities for the occurrence of actual and justified trust. Institutionalising distrust in AI oversight thus aims to increase the trustworthiness of AI governance and to support the emergence of justified trust in AI systems.

Now, what are the principles of democratic rule which institutionalise distrust? Sztompka argues that there are at least twelve: legitimacy; periodical elections and terms of office; majority and collective decisions; division of powers, checks and balances, and limited competence of institutions; rule of law and independent courts; constitutionalism and judicial review; litigation; due process; civic rights; law enforcement; universalism and fairness; open communication (Sztompka 2000: 12–14). For the limited domain of human oversight, we can condense these 12 principles further, as the next sub-section shows.

### Six design principles for human oversight

Drawing on Sztompka’s work, this article suggests six principles to address the challenges to human oversight as typified in the letters A–D in Table 2.

**Table 2:** Challenges to first- and second-degree human oversight

	First-degree oversight	Second-degree oversight
Lack of competence	A	B
Wrong incentives	C	D

*Justification (A, B, C, D)*: Public authority requires justification to be legitimate. Most democratic theorists would agree that democratic procedures such as voting and public deliberation can transfer legitimate authority to holders of public offices. Besides answering the moral question of who has a right to rule, legitimacy also has an institutional dimension. Here, we formulate our expectations in terms of (professional) competence for public institutions and its officials, given the functions we assign them to fulfil (Laux 2022: 7]). A lack of competence of human overseers of AI thus affects the institutional dimension of legitimacy. The principle of justification applies directly to AI oversight in public institutions and for the provision of public services. Scholarship on algorithmic accountability has, however, argued that the need for justification also extends to private institutions, as the designs, operations, and outcomes of automated decision-making systems require justification towards all decision-subjects (Binns 2018: 544).

Green recently suggested that public institutions should be required to write a report in which they justify the implementation of an algorithmic decision-making system. Moreover, whatever form of human oversight is proposed, its functioning needs to be backed by empirical evidence (Green 2022: 12). If missing, such evidence should be produced via “experimental evaluations of human-algorithm collaborations” (Green 2022: 14). In other words, Green requires proof of competence for human oversight to justify the use of AI in government.

There is much to be said in favour of this approach, at least where workable benchmarks are available. While avoiding deaths and injuries should be an uncontroversial safety benchmark, producing evidence that human overseers safeguard fundamental rights to a satisfying degree can suffer from indeterminacy. It requires agreeing on what good normative outcomes are (Elster 2013: 3–4), for example regarding the fairness of algorithmic decisions. Written justifications of using AI and how to oversee the technology must, therefore, beware of creating illusions of normative certainty (cf. also: [Laux et. al 2023a].

The emerging laws of oversight acknowledge the institutional dimension of legitimacy. As shown in Sect. 2, the AIA demands human overseers to be competent (or “AI literate”), well trained, and sufficiently authorised to intervene in an AI system. However, it would be too much to read a requirement to empirically test the effectiveness of human oversight into the AIA proposal. This does not mean though that such testing would not be instrumental for satisfying the principle of justification.

Regarding Table 2, the institutional dimension of legitimacy, therefore, directly addresses areas A and B. Both first- and second-order overseers must reliably perform their tasks—wherever the performance threshold for each in any given AI system may lie. Note that first- and second-degree overseers may require different domains of competence. A doctor working with an AI system to find the best treatment for a patient will obviously have to be both competent in medicine as well as in interacting with a medical AI. An auditor of an AI system used in a hospital may not necessarily need a full medical education but instead advanced statistical training to detect biases in treatment recommendations.

Wrong incentives can likewise obstruct the effectiveness of human oversight and, hence, concern the institutional dimension of legitimacy (areas C and D). How, for example, external incentive structures or intrinsic motivation affect the performance of human overseers requires further research. The principle of justification, therefore, suggests to empirically test the behavioural factors of human oversight (for a behavioural test of legitimacy, see generally: [Laux 2022: 267–287]).

*Periodical mandates (D)*: The AIA requires providers of AI systems to assess conformity either through internal controls or through third-party audits, so-called “notified bodies” (cf. (Mökander et al. 2022: 248–253)). As much as AI developers will be able to select auditors of their choice, implementing a system of rotation for auditors can foster their impartiality by shielding them from being captured by developers’ interests. In an AI economy with particularly strong market actors, external auditors (i.e. second-degree human overseers) may experience the need to play ball with their clients to receive repeat commissions (Laux et. al 2021).

At the same time, periodical mandates may undermine the competence of auditors as they need to acquire skills and experience in vetting AI systems. Institutional design choices thus include trade-offs between principles which institutionalise distrust. While the principle of justification demands competence, the principle of periodical mandates may undermine its emergence. This requires AI governance to find a balance between both, for example by introducing *some* rotation while demanding a certain minimum threshold of competence. Periodical mandates thus address area D and can potentially be detrimental to areas B.

Collective decisions (A*, B*, C, D): Collectivising decisions anticipates that some decision-makers may have harmful motivations. As Braithwaite writes: “When we put twelve citizens on a jury instead of one, through numbers we institutionalize distrust that some jurors are corruptible” (Braithwaite 1998: 369). Article 14(5) AIA stipulates that for high-risk AI systems referred to in point 1(a) of Annex III, actions of decisions need to be “verified and confirmed by at least two natural persons.” The AI systems in question are for remote biometric identification of natural persons. This provision leaves many questions open as to its rationale and scope, as there is no reason given for why for biometrics two human overseers are better than one (Wendehorst 2021: 59, 102–103). We could reconstruct it as an institutional design choice to prevent false incentives from obstructing the oversight of biometric identification.

Taking a positive view, collectives may also improve decisions. Groups can enhance their epistemic competence through diversity (Landemore and Elster 2012; Page 2007); for an application of mechanisms of collective wisdom to the institutional design of EU law, see: [Laux 2022]). Group member’s individual biases may cancel each other out if we aggregate their judgments. By adding members with different life experiences and cognitive problem-solving skills, and given favourable conditions, diverse groups may even outperform experts (Hong and Page 2004). In content moderation, for example, there are attempts to harvest diversity through crowdsourcing (Kyriakou et al. 2021). Meta’s Oversight Board “includes members from a variety of cultural and professional backgrounds”, thus increasing the cognitive diversity of its five-member panels which review and adjudicate cases (for panel size, cf. (Wong and Floridi 2022: 3)). If Art 14(5) AIA were to aim at increasing the competence of oversight through collectivising decisions, it should add a third member, since a two-member group will almost always be too small to aggregate its members’ judgments for epistemic gains (Elster 2013: 41–42).

Collectivising decisions necessitates consulting social choice theory. Decision-making in groups can cascade or become cyclic (Elster 2013: 12; Patty and Penn 2014: 12–35). Groups may become polarised or amplify the individual errors of the group’s members instead of correcting them ((Sunstein and Hastie 2015: 23–24); for an overview, see: [Laux 2022: 138–146]). Collective decisions thus require carefully crafted decision rules concerning, for example, whether to vote by majority or unanimity or whether to aggregate judgments or to argue or bargain over outcomes (Elster 2013: 27–42). Improved competence through collectivising decisions is, therefore, not a guaranteed outcome, hence the asterisks used for areas A and B.

Limited competence of institutions (B*, D): As Sztompka writes, limiting the competence of institutions “implies the suspicion that institutions will tend to expand, monopolize decisions, abuse their powers” (Sztompka 2000: 13). Separation of powers is one of the oldest principles of institutionalising distrust in democratic republics (Braithwaite 1998: 369).

In a layered structure of human oversight, second-degree overseers provide checks on the first-degree overseers. There may be a risk that national competent authorities (Art. 59 AIA) and notified bodies (Art. 33 AIA) could seek to expand their competencies under the AIA. In AI governance, an institution’s ability to expand its powers will likely depend on its technical competence and understanding of AI technology. The better an oversight institution becomes at signalling its technical competence and at catering to the interests of respective audiences, the more other institutions may be inclined to defer to its decisions. Article 37 AIA offers a structured procedure to bring forward challenges to the competence of notified bodies. However, this procedure actualises the principle of justification: As much as legitimate authority requires professional competence, limiting the powers of a competent institution and re-distributing decision-making power to a less competent institution faces justificatory obstacles.

AI governance would thus benefit from the establishment of institutions for second-degree oversight with comparative levels of technical competence, allowing for vertical controls between second-degree overseers. A market-based solution which allows for competition to emerge between oversight providers could prove to be effective. Drawing again on the field of aviation, the different design approaches of soft versus hard automation were developed by two market competitors: Airbus (hard automation) and Boeing (soft automation) rivalled each other as to who develops the best approach (Banks et al. 2019: 256).

Limiting the competence of institutions thus addresses first and foremost area D. In as much as competition between oversight providers is executed on competence, it may have positive effects for area B (hence the asterisk).

*Justiciability and Accountability (A, B, C, D)*: In liberal democracies, individual rights allow citizens to take both private and public institutions (as well as other citizens) to court in defence of their justified claims (Sztompka 2000: 14). The AIA proposal has been criticised for not including procedural rights for individuals to contest automated decisions and seek redress (Smuha et al. 2021: iii). Both the Council and the European Parliament thus proposed to include rights for individuals to lodge complaints with a national supervisory authority (Art. 63(11) AIA*Council; Art. 68a AIA*Parliament). Depending on the final wording of the AIA, individuals adversely affected by an AI system may thus hold ineffective human oversight to account.

Appeals through second-degree oversight offer another avenue for accountability (cf. (Article 29 Data Protection Working Party 2017: 30; Enarsson, et al. 2022). Article 45 AIA demands that Member States “ensure that an appeal procedure against decisions of the notified bodies is available to parties having a legitimate interest in that decision.” Delineating the scope of “legitimate interests” may become a contested issue. The institutional measure of appeals is nevertheless a valuable addition to the governance structure of AI. As mentioned, with its Oversight Board Meta has already implemented an appeals structure in a private institution.

One could further consider whether liability claims against human overseers should be introduced where they do not yet exist in sectoral regulation. First-degree overseers such as doctors are already regularly subject to negligence and malpractice regimes. Appeals procedures and liability regimes could intervene when first- or second-degree human oversight fails out of intent or negligence. In this regard, the European Commission’s 2022 proposals of a novel AI Liability Directive (“AILD”) and a revision of the Product Liability Directive (“PLD”) will be of great importance (European Commission, 2022a, 2022b). However, in its current draft version, the AILD proposal appears to not be applicable in situations in which a human overseer intervenes between the AI output and the damage (Hacker 2022: 18–19).

While appeals of first-degree oversight decisions naturally address areas A and C, liability regimes are conceivable for both second- and first-degree overseers, thus covering all areas of concern from A to D. To be effective, decision-subjects will regularly need to be presented with reasons and explanations of the design and operation of human oversight. Otherwise, they will lack information as to when, why, and how human overseers failed at their task. The principle of justiciability and accountability thus relies on the information revealed through implementing the principle of justification and the principle of transparency introduced below.

*Transparency (A, B, C, D)*: Transparent institutions engender trust (Sztompka 2000: 9). Automated decision-making systems have long been associated with opacity and “black-box” decisions (Burrell 2016; Pasquale 2015). By now, explainable AI and algorithmic transparency have become key mechanisms of AI governance which have been embraced by both policymakers and scholars (Green 2022: 8; Mittelstadt et al. 2019; Wachter et al. 2018). Transparency of the AI’s code, data, and development process are necessary conditions for effective human oversight (Green 2022: 13).

For this article, however, the question is how transparent human oversight itself should be. First, transparency is required as to whether human involvement is real or merely apparent. AI deployers are incentivised to create the appearance of a human-in-the-loop to avoid alienation of some users (Brennan-Marquez et al. 2019: 752). Think of chatbots, for example, that mimic the way humans speak, including pauses and tics, to not give away the impression that they are fully automated agents (Brennan-Marquez et al. 2019: 753–755). There is thus a risk that human trust is being gamed through human-imitating AI. As mentioned, research suggests that humans trust an AI system more if they are told that a human is kept in the loop. Automated assistance devices have been shown to be perceived as more trustworthy if they give the appearance of being human (Metcalf et al. 2019).

Second, if human oversight itself relies to some extent on AI (cf. Section 2), then this fact should be disclosed. Moreover, the methods and data utilised for AI-assisted oversight should also be made transparent. Likewise, the results of the empirical testing of human overseers’ performance as suggested under the principle of justification should be publicly shared, especially when concerning the use of AI in the public sector. The design and operation of human oversight practices should likewise be made public.

Lastly, transparency requires more than the mere provision of information (cf. ([Laux et. al 2023b: 25])). If the public is supposed to have trust in human oversight, transparent information about its procedure and performance needs to be absorbable by laypeople citizens (Mittelstadt et al. 2019). Transparency can then help to mitigate both the challenges of competence and incentives for first- and second-degree human overseers.

### Results

Taken together, the six principles of institutionalised distrust address all areas of concern identified in this article. The principles are interlinked and can reinforce each other, as shown for the principles of transparency and justification. They may also come into conflict. Periodical mandates can help prevent overseers to pursue harmful incentives but may also hinder the emergence of competence which the principle of justification requires. Trade-offs in the realisation of all principles are thus unavoidable, requiring further normative choices. Moreover, there is a benchmark question as to what the institutional design of oversight roles is supposed to achieve. Are we assessing human oversight negatively by its ability to “prevent the prevention” of good outcomes (Elster 2013: 2), for example through removing distorting factors such as biases in their decision-making; or do we assess it positively by its ability to produce good outcomes, for example through increasing competence through collective decisions (for such a positive approach to institutional design, see: [Laux 2022]). A positive approach requires knowing—or deciding—what good outcomes are. Workable benchmarks may be easier to agree on for safety rationales than for value judgments such as those about fairness in automated decision-making.

Table 3 provides an overview of all the expected effects of each principle on each area of concern. Whereas all applicable principles positively address C and D (wrong incentives for first- and second-degree overseers), their influence on areas A and B (lack of competence for first- and second-degree overseers) appears more uncertain. At worst, the effect on competence can even be negative as seen with the introduction of periodical mandates. Moreover, there are no differences between the effects of the six principles on first- and second-degree overseers. This is to be expected for this article’s level of generality. At the local level of a particular AI system, the effects may well diverge between the two types of overseers. The principles of periodical mandates and of limited competence of institutions only apply to second-degree oversight.

**Table 3:** The anticipated effects of the six principles

	A	B	C	D
1. Legitimacy	+	+	+	+
2. Periodical mandates	n/a	–*	n/a	+
3. Collective decisions	+ *	+ *	+	+
4. Limited competence of institutions	n/a	+ *	n/a	+
5. Justiciability and accountability	+	+	+	+
6. Transparency	+	+	+	+

A: first-degree oversight/lack of competence; B: second-degree oversight/lack of competence; C: first-degree oversight/wrong incentives; D: second-degree oversight/wrong incentives. “+” signifies a positive influence; “-” signifies a negative influence; “*” signifies an expected high degree of uncertainty in the outcome

## Conclusion

Utilising distrust for the design of institutions has a long tradition in democratic theory. Applying its tenets to human oversight of AI would render AI governance more trustworthy and, drawing on Sztompka’s paradox of democracy, could generate well-placed trust in AI. The principles suggested in this article provide a scaffolding for the implementation of trustworthy human oversight in local AI systems. Horizontal provisions such as Article 14 AIA will regularly not deliver more than general requirements such as that oversight must be effective. The principle of justification already states that demonstratively ineffective oversight is not trustworthy. Standardisation under the AIA should make the disclosure of information on the performance of human overseers mandatory (for default disclosures in standards, see: [Laux et. al 2023a]). The other five principles will regularly increase the effectiveness of oversight, by boosting competence or by limiting the influence of false incentives.

Moreover, the analysis of the AIA suggests that human oversight is a shared responsibility between AI developers and users. The obligation to execute oversight falls predominantly on users but should include developers for continuously learning AI systems on a case-by-case basis. The taxonomy of oversight roles allows to propose suggestions for improving effectiveness tailored to the type of oversight in question. Norms can be adjusted as to whether oversight needs to be constitutive or corrective of an AI’s output. So far, the emerging laws of oversight do not thoroughly distinguish these types of oversight.

Lastly, AI systems appear to increasingly put human discretion under scrutiny, pressuring humans to justify their performance against the measured outputs of automated decisions. The suggested principles of institutionalised distrust could be read as facilitating a performance-based comparison system between humans and AI. The essence of human oversight, however, lies in maintaining human control of technology. Institutionalising distrust in the abilities and motivations of human overseers is thus only aimed at preventing the worst outcomes and improving overseers’ performance, not at providing arguments for eradicating human control.

## Data Availability

Data sharing not applicable to this article as no datasets were generated or analysed during the current study.

## Footnotes

1 I am thankful to Prof Jeanette Hofmann for directing my attention to Piotr Sztompka’s work. Thank you to Prof Christoph Burchard and the participants of the ConTrust lecture series at the University of Frankfurt for their feedback on an early-stage presentation of the manuscript. I am also indebted to Prof Brent Mittelstadt and Rory Gillis for their comments on an earlier draft. All mistakes are solely those of the author.

2 Cf. also the definition of meaningful human oversight by Brennan-Marquez et al. that tracks the debate around Article 22 GDPR (Brennan-Marquez et al. 2019: 749).

3 On the possible future pathways for standardisation, see Laux et. al (2023a).

4 Cf. https://www.iso.org/committee/6794475.html, last accessed on 24 August 2023.

5 Besides Sztompka’s and Braithwaite’s contributions on institutionalising distrust, cf. the seminal work by Ely (1980) and the contributions in Hardin (2004). On “liberal distrust”, see Hardin (2002). See further the overview in Bertsou (2019: 215–218).
